# Comparison of the ISU, NCI, MSM, and SPADE Methods for Estimating Usual Intake: A Simulation Study of Nutrients Consumed Daily

**DOI:** 10.3390/nu8030166

**Published:** 2016-03-15

**Authors:** Greice H. C. Laureano, Vanessa B. L. Torman, Sandra P. Crispim, Arnold L. M. Dekkers, Suzi A. Camey

**Affiliations:** 1 Post-Graduate Program in Epidemiology, Federal University of Rio Grande do Sul (UFRGS), Porto Alegre 90000-000, Brazil; 2 Post-Graduate Program in Epidemiology and Department of Statistics—UFRGS, Porto Alegre 90000-000, Brazil; vanessa.leotti@ufrgs.br (V.B.L.T.); camey@mat.ufrgs.br (S.A.C.); 3 Department of Nutrition, Federal University of Paraná (UFPR), Curitiba 80000-000, Brazil; crispim@ufpr.br; 4 Netherlands National Institute for Public Health and the Environment, Bilthoven 3720, The Netherlands; arnold.dekkers@rivm.nl

**Keywords:** daily-consumed nutrients distribution, comparison, ISU, NCI, MSM, SPADE

## Abstract

Various methods are available for estimating usual dietary intake distributions. Hence, there is a need for simulation studies to compare them. The methods Iowa State University (ISU), National Cancer Institute (NCI), Multiple Source Method (MSM) and Statistical Program to Assess Dietary Exposure (SPADE) were previously compared in another study, but some results were inconclusive due to the small number of replications used in the simulation. Seeking to overcome this limitation, the present study used 1000 simulated samples for 12 different scenarios to compare the accuracy of estimates yielded by the aforementioned methods. The focus is on scenarios that exhibited the most uncertainty in the conclusions of the mentioned study above, *i.e.*, scenarios with small sample sizes, skewed intake distributions, and large ratios of the between- and within-person variances. Bias was used as a measure of accuracy. For scenarios with small sample sizes (n=150), the ISU, MSM and SPADE methods generally achieved more accurate estimates than the NCI method, particularly for the 10th and 90th percentiles. The differences between methods became smaller with larger sample sizes (*n* = 300 and *n* = 500). With few exceptions, the methods were found to perform similarly.

## 1. Introduction

The assessment of usual dietary intake (*i.e.*, long-term average intake) is a topic of current interest in the field of nutrition, as many diseases are influenced or even caused by individual dietary habits [1]. In particular, the study of usual intake distributions can help us to identify population groups who are at risk of having an inadequate dietary intake, either for insufficient or excessive consumption. The methods that are currently applied for estimating usual intake distributions use data that assess dietary intake over at least two independent days for each subject. It bears stressing that there is no gold-standard method for dietary intake assessment, although the most widely used include 24-h dietary recalls (24-HDRs), food frequency questionnaires (FFQs), and dietary records. However, when assessing the long-term average intake from short-term measurements, the data derived from such measurements require statistical modeling in order to take into account between-person and within-person variations. The main reason for using statistical modeling for estimating usual intake distributions is to handle skewed data and to distinguish and remove the day-to-day (short-term) variation (within-person variation) from the total variation.

Various statistical methodologies have been proposed for estimating usual intake distributions [2,3,4,5,6,7,8,9,10,11,12,13,14,15,16,17,18,19,20,21]. Although the same general approach is used, the methods may differ when it comes to details such as the numerical methods used, software implementation, or differences in underlying assumptions. In this regard, some questions are still unanswered in the literature: What is the accuracy and precision of such methods? How should they be compared with real or simulated data? Some efforts have been made in this direction [6,7,9,16,19,21,22,23].

Souverein *et al.* [22] compared the Iowa State University (ISU), the National Cancer Institute (NCI), the Multiple Source Method (MSM) and the Statistical Program to Assess Dietary Exposure (SPADE) methods by assessing the influence of sample size, ratio of the within- and between-person variances, and Box-Cox transformation parameter values on the quality of their estimates [24]. Souverein *et al.* [22] concluded that the various methods generated similar estimates for most scenarios, but estimates diverged and bias increased when the variance ratio increased above 4 and the sample size decreased below 500. However, this study used only 100 replicates per scenario and used three samples sizes, which can be considered somewhat extreme. In fact, an intermediate sample size (between 150 and 500) should be more interesting because results were consistent for sample sizes greater than 500. These limitations prevented solid conclusions from being drawn as to the quality of the methods tested in some scenarios and percentiles, particularly the 90th percentile.

Therefore, the present paper reports on a simulation study conducted using the same approach as Souverein *et al.* [22], comparing once again the ISU, NCI, MSM and SPADE methods for daily-consumed nutrient intakes. Our study was focused on those scenarios that exhibited the most uncertainty in the conclusions of Souverein *et al.* [22]. We used a greater number of replicates (1000), small to moderate sample sizes (n = 150, 300 and 500), and diversified the values of the within- and between-person variances in the simulation, with large ratios of the between- and within-person variances.

## 2. Materials and Methods 

The ISU method was proposed by the Iowa State University [6,7,18] and has two different implementations: one in SAS [25], which was used in this study, and a menu driven stand-alone version, which can be obtained from the authors upon request at the ISU-SIDE website [26]. To estimate the usual intake distribution for daily-consumed nutrients, the ISU method follows four steps:

1. The ratio of the shifted, power-transformed observed intakes is adjusted to take into account nuisance effects, such as day of the week and interview mode (telephone or in-person). Construct smoothed daily intakes by undoing the initial power transformation and shifting for the adjusted observations.
2. A grafted polynomial function is fit to the normal probability plot of the smoothed intakes using least-squares. The inverse of the fitted function is used to transform the smoothed intakes to normality.
3. Moment estimates of variance components are computed for the transformed intakes, and an estimate of the normal-scale usual intake distribution is obtained,
4. A grafted cubic and a 9-point approximation to use to transform the normal-scale usual intake distribution to original scale.

The NCI method, as its name implies, was proposed by the U.S. National Cancer Institute [10,11,15,16]. It has been implemented with SAS macros [25] and is available [27]. In this study, version 2.1 of the SAS macros was used. To estimate the usual intake distribution of daily-consumed nutrients, the NCI method follows four steps:

1. The observed intakes are transformed to improve normality by means of a one-parameter Box-Cox transformation, indicated by λ in this paper.
2. A linear mixed effects model on the transformed intake data is fit to estimate the mean and the within- and between-person variances.
3. k (value to be set) pseudo-person intakes from a normal distribution is simulated with mean equal to the estimated mean and variance equal to the between-person variance.
4. The simulated values by a 9-point approximation is back-transformed, which involves the estimated Box-Cox parameter and the within-person variation.

The MSM was proposed for use in Europe by a German team [17,28] within the European Food Consumption Validation (EFCOVAL) consortium and is available through an online interface [29]. To estimate the usual intake distribution for daily-consumed nutrients, the MSM method proposes five steps:

1. A linear regression model is applied to the data and the residuals are used for the shrinkage part of the MSM method.
2. The fitted model residuals are transformed to normality by means of a two-parameter Box-Cox transformation, with λ restricted to 1/λ=1,2,3….
3. The within- and between-person variances are estimated by means of the transformed residuals.
4. The back-transformation is defined by a closed formula, involving the estimated λ and the within-person variance.
5. The distribution is estimated by the inverse regression model after the back-transformation to the original scale of the residuals.

The SPADE [19,21,22] method is implemented in R software [30] and is based on the AGEMODE [13] model, where intake estimates are modeled with age as a covariate. However, although SPADE considers the model as a function of age, this information can be omitted after minor adjustments to the software, which enables the comparison with the other methods. SPADE is freely available as an R-package called SPADE.RIVM [31]. To estimate the usual intake distribution for daily-consumed nutrients, the SPADE method follows four steps:

1. The observed intakes are transformed by means of a one-parameter Box-Cox transformation.
2. A linear mixed effects model on the transformed scale is used to estimate the mean and within-person and between-person variances.
3. The mean on the transformed scale is directly back-transformed by Gaussian Quadrature, using the total variance of the model and the Box-Cox transformation parameter λ.
4. The percentiles on the transformed scale correspond exactly with the percentiles on the original scale, and their back-transformation by Gaussian Quadrature involves the within-person variance and λ [19]. The distribution is calculated directly in the back-transformation step.

### Simulations

Data simulation was used for the intake of daily-consumed nutrients with a Box-Cox distribution. For this purpose, we defined the following parameters on the transformed scale: overall mean intake (μ), between-person standard deviation ($\sigma_{u}$), within-person standard deviation ($\sigma_{\varepsilon }$), the ratio of within- and between-person variances $\left(r_{var}=\left(\sigma_{\varepsilon }^{2}/\sigma_{u}^{2}\right)\right)$, and the Box-Cox transformation parameter (λ).

Twelve scenarios were generated based on the simulation results of Souverein *et al.* [22]. We explored the scenarios that had the most uncertainty in the results of this study, including sample sizes of 150, 300 and 500 and $r_{var}$ values of 4 and 9. Because Souverein *et al.* [22] did not provide the values for the variances, we decided to use different combinations of variance values. Box 1 shows the chosen parameter values for each scenario.

Box 1 **Simulation scenarios.**

**Scenario*****n*****Within-Person Variance**
$\left(\sigma_{\varepsilon }^{2}\right)$**Between-Person Variance**$\;\left(\sigma_{u}^{2}\right)$**Variance Ratio**
$\left(r_{var}\right)$I15010.254II0.119III3000.254IV0.119V5000.254VI0.119VII1501.20.34VIII2.79IX3001.24X2.79XI5001.24XII2.79

Souverein *et al.* [21] discussed in their study that, although an $r_{var}$ equal or higher to 9 is rare, there are cases where this has been reported [1,32] for nutrients: zinc in women, vitamin B-12 in men, and vitamin A in women and men.

The simulated data for intake of daily-consumed nutrients were generated as follows:

First, we generated for each scenario *n* individual means from a normal distribution with mean μ=7.5 and the between-person variance as described in Box 1. We then generated two daily intake observations per subject on the transformed scale, using a normal distribution with the individual mean intake generated in the previous step and the within-person variance as described in Box 1. Finally, we applied the Box-Cox back-transformation (λ=0.2) to transform the two intakes back to the original scale. These definitions generated a mean intake on the original scale equal to 105.56 for scenarios I, III, and V, equal to 104.67 for scenarios II, IV, and VI, equal to 107.17 for scenarios VII, IX, and XI, and equal to 116.95 for scenarios VIII, X, and XII.

The software environments employed for simulation were R to generate data and run the SPADE method, SAS to run the ISU and NCI methods, and AutoHotkey [33] to automate the MSM method.

To compare estimates, we calculated mean bias B for each method,

(1) $$B\left(\hat{\theta }\right)=\frac{\sum_{i=1}^{N}\left({\hat{\theta }}_{j}-\theta \right)}{N}$$

relative (percent) bias RB,

(2) $$\text{RB}\left(\hat{\theta }\right)=\left|\frac{B\left(\hat{\theta }\right)}{\theta }\right|\times 100$$

and mean squared error MSE,

(3) $$\text{MSE}\left(\hat{\theta }\right)=\frac{\sum_{i=1}^{N}{\left({\hat{\theta }}_{j}-\theta \right)}^{2}}{N}$$

where ${\hat{\theta }}_{j}$ is the estimated value of the parameter for the replicate j, θ is the true value of the parameter, and N is the number of replicates in the simulation. Bonferroni confidence intervals for the mean bias with 95% confidence level were also calculated to compare the methods.

To calculate these measures, we calculated the true mean and percentiles through Gaussian quadrature (obtained with the *f.gauss.quad* function implemented in the R-library SPADE.RIVM). This function enables the calculation of the mean and percentiles on the original scale using the parameters μ, the between- and within-person variances (all in transformed scale), and λ, if all the model assumptions are fulfilled [19].

The code for generating the data is provided as supplementary material to this article.

## 3. Results

In this study, the ISU, NCI and SPADE methods were not able to yield estimates for some simulated samples, per scenario. When the ISU and NCI methods estimated the between-person variance as zero, they were unable to complete the estimations. SPADE completed the estimations, but all percentiles were equal, indicating an estimated between-person variance equal to zero. When this happened with at least one of the methods, the sample was excluded from the analysis for all methods.

**Figure 1:**
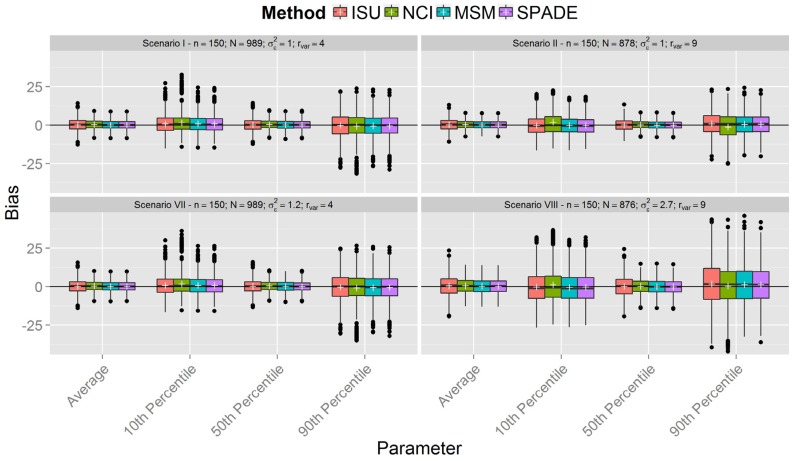
Boxplot of biases calculated for each method and scenarios with *n* = 150 for samples with estimated between-person variance different from zero for all methods (N denotes the number of usable samples).

**Figure 2:**
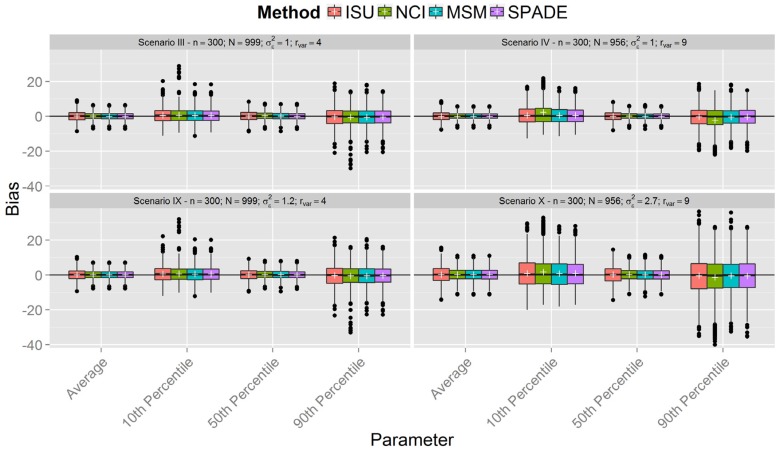
Boxplot of biases calculated for each method and scenarios with *n* = 300 for samples with estimated between-person variance different from zero for all methods (N denotes the number of usable samples).

**Figure 3:**
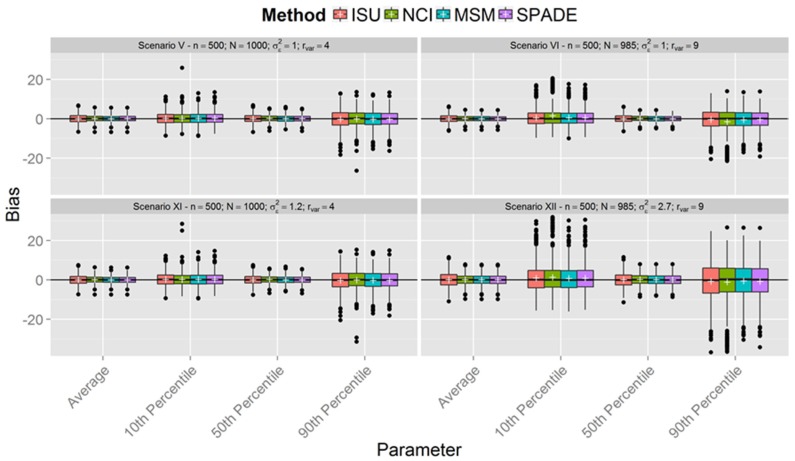
Boxplot of biases calculated for each method and scenarios with *n* = 500 for samples with estimated between-person variance different from zero for all methods (N denotes the number of usable samples).

Figure 1, Figure 2 and Figure 3 show boxplots of the biases in each scenario with sample sizes of 150, 300, and 500 respectively, confirming similar results between the methods. However, it is clear that all methods were less accurate for estimation of the 10th and 90th percentiles across all scenarios. As expected, accuracy was lower in scenarios with a smaller sample size (*n* = 150). It is interesting to note that, in the first six scenarios with between-person variance equal to one (the upper plots in Figure 1, Figure 2 and Figure 3), the differences in the spread of the bias are much fewer than in the scenarios VII-XII (the lower plots in Figure 1, Figure 2 and Figure 3).

**Figure 4:**
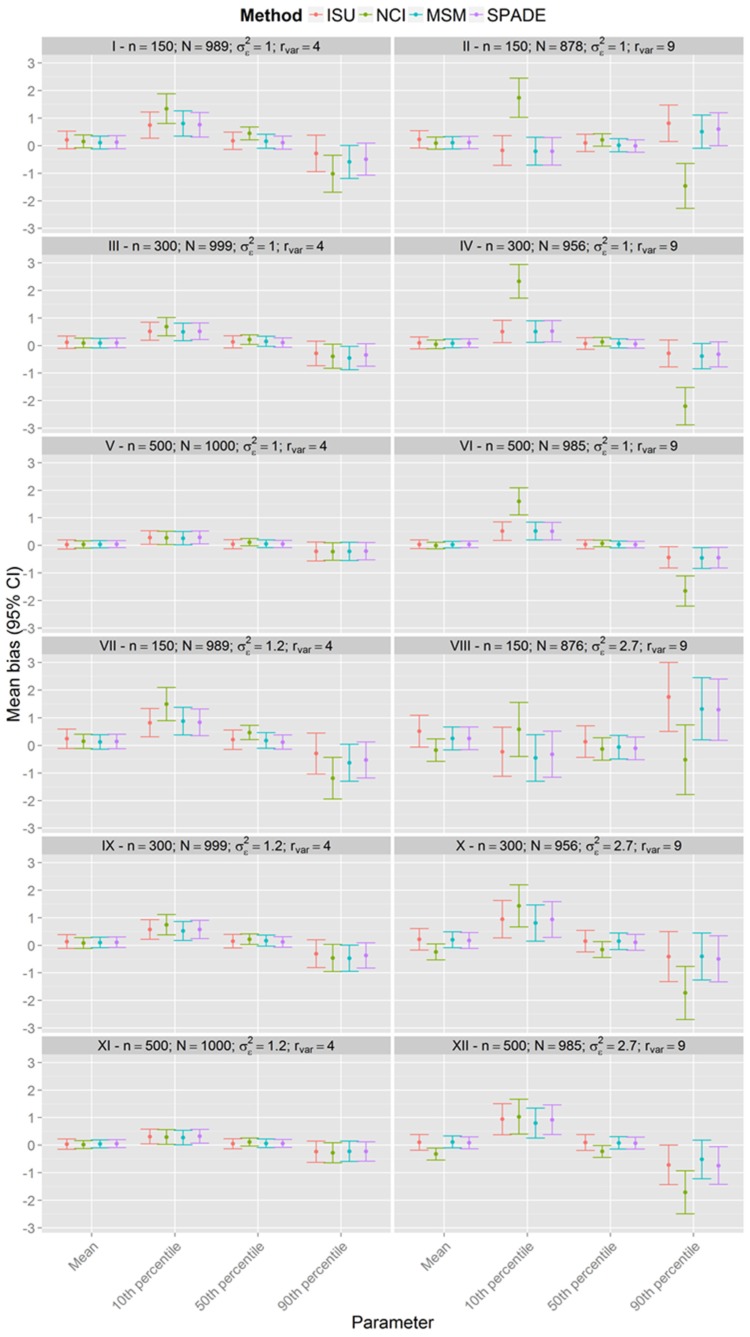
Bonferroni confidence interval for the mean bias with 95% confidence level for each scenario for samples with estimated between-person variance different from zero for all methods (n indicates the sample size and N the number of usable simulated samples).

Figure 1, Figure 2 and Figure 3 show that the methods tend to overestimate the 10th percentile and to underestimate the 90th percentile, indicating a greater shrinkage of the data than expected. Figure 4 shows that the biases for the mean and the median are not statistically significant, except for the NCI method in scenario XII. There is a statistically significant overestimation of the 10th percentile in all methods except in scenarios II and VIII, whereas the 90th percentile is sometimes overestimated (scenarios II and VIII) and underestimated to a statistically significant degree by all methods in scenario VI. The NCI method showed a statistically significant larger bias than the ISU, SPADE, and MSM methods for the percentiles 10th and 90th in scenarios II, IV, and VI.

More results are presented in the supplementary material, including: the number of simulated samples for which the between-person variance was estimated as zero; the bias and relative bias of estimates in the 5th, 10th, 25th, 50th, 75th, 90th, and 95th percentiles for each scenario; the Mean Square Error (MSE) of estimates for each scenario; the boxplot of biases calculated for each method with more percentiles; as well as two tables with the bias (relative bias) and the MSE for each method with all available results—without excluding samples when other methods estimated between-person variance equal to zero.

## 4. Discussion

This paper reports the results of a simulation study that compared four methods employed for estimation of usual dietary intake distributions of daily-consumed nutrients, suggesting that, with a few exceptions, they performed similarly. The results obtained from the simulated scenarios showed that the bias of estimated mean and percentiles of all methods decreased when sample sizes increased and the ratio of variances was fixed. Furthermore, the ratio of variances had a small impact on the bias of the mean and median, although the variation in the bias increased for larger ratio of variances in the last six scenarios (compare in Figure 1, Figure 2 and Figure 3 each lower plot, representing one of the last six scenarios, with the plot above). Other percentiles showed a larger bias for larger ratios. Indeed, the further the percentiles are from the median, the larger the biases.

Results also showed a poorer quality of estimation with all methods with respect to the 10th and 90th percentiles. Since one of the interests of estimating usual intake distributions in a population is to assess whether they have nutritional inadequacies in deficit or excess [34], a valid estimation of these percentiles is of the utmost importance and of concern.

In terms of accuracy, all four methods were similar with relatively low bias, but the behavior of the methods was different for the estimation of the mean usual intake and for the estimation of the percentiles. The methods use different numerical procedures to estimate the within- and between-person variances, which may cause numerical problems like an estimated between-person variance close to zero. These estimated values lead to unusable results such as unrealistically small differences between the estimated 10th and 90th percentiles. This happened for all methods, except the MSM.

In most of the scenarios, all methods seemed to shrink the intake distributions more than expected, resulting in overestimation of the low percentiles and underestimation of the high percentiles. For the NCI, when the sample size was small and the ratio was greater, the within-person variance seemed to be overestimated. This probably resulted in shrinkage greater than expected, which can be seen in the estimates of the percentiles. In fact, the NCI method showed only comparable results for scenarios V and XI, with *n* = 500 and$\;r_{var}=4$.

The behavior of the MSM and SPADE methods was similar for almost all scenarios. ISU seemed to perform better for scenarios I and VII and worse for II and VIII compared to MSM and SPADE for higher percentiles. This may indicate that, for lower ratio values, the ISU method is better than MSM and SPADE for small sample sizes, but worse for small sample sizes (*n* = 150) and a higher ratio. These differences disappeared for simulations with *n* = 300 and *n* = 500.

It is noteworthy that the NCI method had larger or equal bias compared to the other methods for the estimate of the mean habitual intake in all scenarios. When the within-person variance is larger than the between-person variance, Tooze *et al.* [16] advise that the NCI method should use the same back-transformation used by the ISU method. In this paper, we used version 2.1 of the NCI with ISU back-transformation implemented (Bethesda, MD, USA); however, as was the case for Souverein *et al.* [22], the NCI method had the worst results in scenarios where the ratio-variance was equal to 9.

It bears stressing that this study did not address the influence of covariates or episodically consumed foods, as well as all possible combinations of sample sizes and parameters that could relate to existent daily consumed nutrients of different populations. For that, the results may differ as it depends on other aspects of the diet. However, further studies are needed to draw any conclusions on this matter.

In this study, a similar approach to the one reported by Souverein *et al.* [22] was proposed, but with a larger number of replications, a greater sample size, and some extra statistics for checking the results. As we used a larger number of replications, the results showed that unstable behavior of estimations not only happened because of the number of replications, but also depended on the sample size and the variance ratio.

## 5. Conclusions

In conclusion, this study showed the importance of the sample size and variance ratio for the quality of the estimation of usual intake distributions of daily consumed nutrients. It showed some limitations to the numerical solutions used in the various methods. Furthermore, the models almost behaved the same, as shown by Souverein *et al.* [22] and Dekkers *et al.* [19,20,21], but the NCI was less accurate for sample sizes of 150 and 300 than the other three methods. We agree with Souverein *et al.* [22] that people can choose their favorite method for practical reasons such as user-friendliness or assessment of the results for making plots, simulations, or a bootstrap. However, we also recommend that, in the case of small sample sizes and/or large within- and between-person variances, one should also use the SPADE or MSM methods to corroborate the results.

## Supplementary Materials

The following are available online at http://www.mdpi.com/2072-6643/8/3/166/s1, Table S1: the number of simulated samples for which the between-person variance was estimated as zero; Table S2: Bias and relative bias of estimates obtained with each method for each scenario (λ=0.2); Table S3**:** MSEs of estimates obtained with each method for each scenario (λ=0.2) and Figure S1: Boxplot of biases calculated for each method and scenario, all results for the methods that had a positive estimate for the between-person variance. Table S4: Bias and relative bias of estimates obtained with each method for each scenario (λ=0.2) and Table S5: MSEs of estimates obtained with each method for each scenario (λ=0.2), both for each method without excluded samples when other methods estimated between-person variance equal to zero. All supplementary tables and figures had more percentile results than in the article (5th, 25th, 75th, and 95th).
