# Supplementary material for: Comparison of the ISU, NCI, MSM, and SPADE Methods for Estimating Usual Intake: A Simulation Study of Nutrients Consumed Daily
Source: Nutrients. 2016 Mar 15;8(3):166. doi: 10.3390/nu8030166 (PMC4808894; doi:10.3390/nu8030166)
Supplement: Supplementary file 1 [file nutrients-08-00166-s001.docx]

**Supplementary Materials: Comparison of the ISU, NCI, MSM, and SPADE Methods for Estimating Usual Intake: A Simulation Study of Nutrients Consumed Daily**

Greice H. C. Laureano, Vanessa B. L. Torman, Sandra P. Crispim, Arnold L. M. Dekkers
and Suzi A. Camey

1. **Data Generation**

# Packages of R

require(lme4)

require(car)

### Initial information

## Data generation for the scenario V ##

sub <- 500 # number of subjects

av <-7.5 # average of intake

var.w <- 1 # within-person variance

rvar <- 4 # variance ratio value

var.b <- var.w/rvar # between-person variance

sd.w <- sqrt(var.w) # within-person standard deviation

sd.b <-sqrt(var.b) # between-person standard deviation

nmax <- sub*2 # number of observations

# Number of repetitions (n° 24-HDRs)

nrep <- 2

# lambda value for the Box- Cox transformation

lambda <- 0.2

### Random effects

set.seed(12356780) # simulation seed

sub_ef = rnorm(sub,0,sd.b) # Random effect

### Generating intake

average = av + sub_ef # Generating average #individual with random effect

sub_ef_rep = rep(average,each=nrep) # effect of subject

### Generating two intakes for each subject

x = rnorm(nmax,sub_ef_rep,sd.w)

### Transforming the data in the Box- Cox scale

x2 <-(((lambda*x)+1)^(1/lambda))

1. **Number of Simulated Samples for Which the Between-Person Variance was Estimated
   as Zero**

Table S1 shows how many times the methods ISU, NCI, and SPADE were unable to yield estimates for some simulated samples, per scenario. When the ISU and NCI methods estimated the between-person variance as zero, they were unable to complete the estimations. SPADE completed the estimations, but all percentiles were equal, indicating an estimated between-person variance equal to zero. Due to this issue, the results reported herein refer to the replicates for which all methods had a positive estimate for the between-person variance. If this happened with at least one of the methods, the sample was excluded from the analysis of all methods.

**Table S1.** Number of simulated samples for which the between-person variance was estimated
as zero.

| **Scenario** | **Method** | | |
| --- | --- | --- | --- |
|  | **ISU** | **NCI** | **SPADE** |
| I | 11 | 1 | 4 |
| II | 122 | 11 | 47 |
| III | 1 | 0 | 0 |
| IV | 44 | 1 | 16 |
| V | 0 | 0 | 0 |
| VI | 15 | 1 | 3 |
| VII | 11 | 0 | 5 |
| VIII | 124 | 0 | 49 |
| IX | 1 | 0 | 0 |
| X | 44 | 0 | 16 |
| XI | 0 | 0 | 0 |
| XII | 15 | 0 | 5 |

ISU, Iowa State University; NCI, National Cancer Institute; MSM, Multiple Source Method; SPADE, Statistical Program to Assess Dietary Exposure.

1. **Results with Samples Excluded When at Least one Method Estimated Between-Person Variance Equal to Zero**

To compare estimates, we calculated, for each method, bias B,

| $B(\hat{\theta})=\frac{\sum_{i=1}^{N} \left( \hat{\theta}_{j}-\theta\right)}{N}$ | (1) |
| --- | --- |

relative (percent) bias RB,

| $RB(\hat{\theta})=\left\vert\frac{B(\hat{\theta})}{\theta} \right\vert\times100$ | (2) |
| --- | --- |

and mean squared error MSE,

| $MSE\left( \hat{\theta} \right)=\frac{\sum_{i=1}^{N} \left( \hat{\theta}_{j}-\theta\right)^{2}}{N}$ | (3) |
| --- | --- |

where $\hat{\theta}_{j}$ is the estimated value of the parameter for the replicate $j$,$\theta$ is the true value of the parameter, and $N$is the number of replicates in the simulation.

Table S2 shows the true value, bias, and relative bias for the mean and some percentiles of the usual intake distribution in each evaluated scenario. The compared methods behaved similarly, yielding estimates close to the true values. Over all scenarios and methods, the range of the relative bias for the mean of the usual intake distributions varied from $0.03\%$ to $0.44\%$, and the corresponding ranges of the 5th and 95th percentiles from $0.18\%$ to $3.41\%$ and from $0.20\%$ to $2.21\%$, respectively.

**Table S2.** Bias and relative bias of estimates obtained with each method for each scenario $\left( \lambda=0.2 \right)$.

| **Scenario** | **Parameter** | **True Value** | **Bias (Relative Bias %)** | | | | | | | |
| --- | --- | --- | --- | --- | --- | --- | --- | --- | --- | --- |
|  |  |  | **ISU** | | **NCI** | | **MSM** | | **SPADE** | |
| I ($\sigma_{\varepsilon}^{2}=1, n = 150$ and $r_{var}=4$) | Average | 105.56 | 0.21 | (0.20) | 0.35 | (0.33) | 0.11 | (0.11) | 0.13 | (0.12) |
|  | 5th Percentile | 74.64 | 0.91 | (1.21) | 1.76 | (2.36) | 0.98 | (1.31) | 0.94 | (1.26) |
|  | 10th Percentile | 80.46 | 0.74 | (0.92) | 1.57 | (1.95) | 0.81 | (1.00) | 0.76 | (0.95) |
|  | 25th Percentile | 90.99 | 0.47 | (0.52) | 0.90 | (0.99) | 0.45 | (0.49) | 0.45 | (0.50) |
|  | 50th Percentile | 103.97 | 0.18 | (0.17) | 0.43 | (0.41) | 0.16 | (0.15) | 0.11 | (0.10) |
|  | 75th Percentile | 118.39 | −0.09 | (0.07) | −0.31 | (0.26) | −0.27 | (0.23) | −0.22 | (0.19) |
|  | 90th Percentile | 132.71 | −0.28 | (0.21) | −0.67 | (0.51) | −0.59 | (0.44) | −0.49 | (0.37) |
|  | 95th Percentile | 141.93 | −0.37 | (0.26) | −0.72 | (0.51) | −0.61 | (0.43) | −0.63 | (0.44) |
| II ($\sigma_{\varepsilon}^{2}=1, n = 150$ and $r_{var}=9$) | Average | 104.67 | 0.23 | (0.22) | 0.24 | (0.23) | 0.11 | (0.10) | 0.12 | (0.11) |
|  | 5th Percentile | 83.56 | −0.19 | (0.23) | 2.35 | (2.82) | −0.15 | (0.18) | −0.20 | (0.24) |
|  | 10th Percentile | 87.76 | −0.17 | (0.20) | 1.92 | (2.19) | −0.20 | (0.23) | −0.21 | (0.23) |
|  | 25th Percentile | 95.16 | −0.09 | (0.10) | 1.01 | (1.06) | −0.19 | (0.20) | −0.16 | (0.16) |
|  | 50th Percentile | 103.97 | 0.10 | (0.10) | 0.23 | (0.22) | 0.02 | (0.02) | −0.01 | (0.01) |
|  | 75th Percentile | 113.41 | 0.41 | (0.36) | −0.66 | (0.58) | 0.24 | (0.21) | 0.25 | (0.22) |
|  | 90th Percentile | 122.50 | 0.81 | (0.66) | −1.22 | (0.99) | 0.51 | (0.42) | 0.60 | (0.49) |
|  | 95th Percentile | 128.21 | 1.12 | (0.87) | −1.43 | (1.11) | 0.84 | (0.65) | 0.87 | (0.68) |
| III ($\sigma_{\varepsilon}^{2}=1, n = 300$ and $r_{var}=4$) | Average | 105.56 | 0.12 | (0.11) | 0.13 | (0.12) | 0.09 | (0.08) | 0.10 | (0.09) |
|  | 5th Percentile | 74.64 | 0.62 | (0.82) | 0.63 | (0.84) | 0.58 | (0.78) | 0.62 | (0.83) |
|  | 10th Percentile | 80.46 | 0.52 | (0.64) | 0.61 | (0.76) | 0.49 | (0.61) | 0.52 | (0.64) |
|  | 25th Percentile | 90.99 | 0.34 | (0.37) | 0.46 | (0.50) | 0.35 | (0.39) | 0.33 | (0.36) |
|  | 50th Percentile | 103.97 | 0.13 | (0.13) | 0.25 | (0.24) | 0.15 | (0.15) | 0.11 | (0.10) |
|  | 75th Percentile | 118.39 | −0.09 | (0.07) | −0.17 | (0.14) | −0.12 | (0.10) | −0.13 | (0.11) |
|  | 90th Percentile | 132.71 | −0.29 | (0.22) | −0.45 | (0.34) | −0.45 | (0.34) | −0.34 | (0.26) |
|  | 95th Percentile | 141.93 | −0.40 | (0.28) | −0.54 | (0.38) | −0.43 | (0.30) | −0.47 | (0.33) |
| IV ($\sigma_{\varepsilon}^{2}=1, n = 300$ and $r_{var}=9$) | Average | 104.67 | 0.09 | (0.09) | 0.09 | (0.09) | 0.08 | (0.08) | 0.08 | (0.08) |
|  | 5th Percentile | 83.56 | 0.64 | (0.77) | 2.85 | (3.41) | 0.65 | (0.78) | 0.66 | (0.79) |
|  | 10th Percentile | 87.76 | 0.51 | (0.58) | 2.31 | (2.64) | 0.51 | (0.58) | 0.52 | (0.59) |
|  | 25th Percentile | 95.16 | 0.29 | (0.31) | 1.33 | (1.39) | 0.33 | (0.34) | 0.29 | (0.31) |
|  | 50th Percentile | 103.97 | 0.07 | (0.07) | 0.19 | (0.18) | 0.08 | (0.07) | 0.06 | (0.06) |
|  | 75th Percentile | 113.41 | −0.13 | (0.11) | −1.09 | (0.96) | −0.18 | (0.16) | −0.15 | (0.14) |
|  | 90th Percentile | 122.50 | −0.28 | (0.23) | −2.20 | (1.80) | −0.39 | (0.32) | −0.32 | (0.26) |
|  | 95th Percentile | 128.21 | −0.36 | (0.28) | −2.83 | (2.21) | −0.39 | (0.30) | −0.41 | (0.32) |
| V ($\sigma_{\varepsilon}^{2}=1, n = 500$ and $r_{var}=4$) | Average | 105.56 | 0.03 | (0.03) | 0.11 | (0.1) | 0.04 | (0.04) | 0.04 | (0.04) |
|  | 5th Percentile | 74.64 | 0.34 | (0.45) | 0.25 | (0.34) | 0.31 | (0.41) | 0.35 | (0.47) |
|  | 10th Percentile | 80.46 | 0.28 | (0.35) | 0.22 | (0.28) | 0.26 | (0.32) | 0.29 | (0.36) |
|  | 25th Percentile | 90.99 | 0.17 | (0.19) | 0.19 | (0.21) | 0.18 | (0.20) | 0.18 | (0.2) |
|  | 50th Percentile | 103.97 | 0.04 | (0.04) | 0.20 | (0.19) | 0.06 | (0.05) | 0.05 | (0.05) |
|  | 75th Percentile | 118.39 | −0.09 | (0.08) | −0.04 | (0.03) | −0.06 | (0.05) | −0.09 | (0.07) |
|  | 90th Percentile | 132.71 | −0.22 | (0.17) | 0.00 | (0.00) | −0.22 | (0.17) | −0.21 | (0.16) |
|  | 95th Percentile | 141.93 | −0.29 | (0.21) | −0.06 | (0.04) | −0.34 | (0.24) | −0.28 | (0.20) |
| VI ($\sigma_{\varepsilon}^{2}=1, n = 500$ and $r_{var}=9$) | Average | 104.67 | 0.04 | (0.04) | 0.06 | (0.06) | 0.03 | (0.03) | 0.03 | (0.03) |
|  | 5th Percentile | 83.56 | 0.65 | (0.78) | 2.00 | (2.40) | 0.63 | (0.75) | 0.65 | (0.78) |
|  | 10th Percentile | 87.76 | 0.52 | (0.59) | 1.59 | (1.82) | 0.52 | (0.59) | 0.52 | (0.59) |
|  | 25th Percentile | 95.16 | 0.29 | (0.30) | 0.90 | (0.95) | 0.29 | (0.30) | 0.29 | (0.30) |
|  | 50th Percentile | 103.97 | 0.04 | (0.03) | 0.14 | (0.14) | 0.03 | (0.03) | 0.03 | (0.03) |
|  | 75th Percentile | 113.41 | −0.22 | (0.19) | −0.79 | (0.70) | −0.21 | (0.18) | −0.22 | (0.20) |
|  | 90th Percentile | 122.50 | −0.44 | (0.36) | −1.51 | (1.23) | −0.46 | (0.37) | −0.44 | (0.36) |
|  | 95th Percentile | 128.21 | −0.56 | (0.44) | −1.99 | (1.55) | −0.61 | (0.47) | −0.57 | (0.45) |
| VII ($\sigma_{u}^{2}=0.3 , n = 150$ and $r_{var}=4$) | Average | 107.17 | 0.21 | (0.20) | 0.35 | (0.33) | 0.11 | (0.11) | 0.13 | (0.12) |
|  | 5th Percentile | 73.26 | 0.91 | (1.21) | 1.76 | (2.36) | 0.98 | (1.31) | 0.94 | (1.26) |
|  | 10th Percentile | 79.54 | 0.74 | (0.92) | 1.57 | (1.95) | 0.81 | (1.00) | 0.76 | (0.95) |
|  | 25th Percentile | 90.99 | 0.47 | (0.52) | 0.90 | (0.99) | 0.45 | (0.49) | 0.45 | (0.50) |
|  | 50th Percentile | 105.24 | 0.18 | (0.17) | 0.43 | (0.41) | 0.16 | (0.15) | 0.11 | (0.10) |
|  | 75th Percentile | 121.24 | −0.09 | (0.07) | −0.31 | (0.26) | −0.27 | (0.23) | −0.22 | (0.19) |
|  | 90th Percentile | 137.26 | −0.28 | (0.21) | −0.67 | (0.51) | −0.59 | (0.44) | −0.49 | (0.37) |
|  | 95th Percentile | 147.63 | −0.37 | (0.26) | −0.72 | (0.51) | −0.61 | (0.43) | −0.63 | (0.44) |

**Table S2.** *Cont*.

| **Scenario** | **Parameter** | **True Value** | **Bias (Relative Bias %)** | | | | | | | |
| --- | --- | --- | --- | --- | --- | --- | --- | --- | --- | --- |
|  |  |  | **ISU** | | **NCI** | | **MSM** | | **SPADE** | |
| VIII ($\sigma_{u}^{2}=0.3, n = 150$ and $r_{var}=9$) | Average | 116.95 | 0.23 | (0.22) | 0.24 | (0.23) | 0.11 | (0.1) | 0.12 | (0.11) |
|  | 5th Percentile | 81.07 | −0.19 | (0.23) | 2.35 | (2.82) | −0.15 | (0.18) | −0.20 | (0.24) |
|  | 10th Percentile | 87.75 | −0.17 | (0.20) | 1.92 | (2.19) | −0.20 | (0.23) | −0.21 | (0.23) |
|  | 25th Percentile | 99.90 | −0.09 | (0.10) | 1.01 | (1.06) | −0.19 | (0.20) | −0.16 | (0.16) |
|  | 50th Percentile | 114.97 | 0.10 | (0.10) | 0.23 | (0.22) | 0.02 | (0.02) | −0.01 | (0.01) |
|  | 75th Percentile | 131.83 | 0.41 | (0.36) | −0.66 | (0.58) | 0.24 | (0.21) | 0.25 | (0.22) |
|  | 90th Percentile | 148.68 | 0.81 | (0.66) | −1.22 | (0.99) | 0.51 | (0.42) | 0.60 | (0.49) |
|  | 95th Percentile | 159.56 | 1.12 | (0.87) | −1.43 | (1.11) | 0.84 | (0.65) | 0.87 | (0.68) |
| IX ($\sigma_{u}^{2}=0.3, n = 300$ and $r_{var}=4$) | Average | 107.17 | 0.12 | (0.11) | 0.13 | (0.12) | 0.09 | (0.08) | 0.10 | (0.09) |
|  | 5th Percentile | 73.26 | 0.62 | (0.82) | 0.63 | (0.84) | 0.58 | (0.78) | 0.62 | (0.83) |
|  | 10th Percentile | 79.54 | 0.52 | (0.64) | 0.61 | (0.76) | 0.49 | (0.61) | 0.52 | (0.64) |
|  | 25th Percentile | 90.99 | 0.34 | (0.37) | 0.46 | (0.50) | 0.35 | (0.39) | 0.33 | (0.36) |
|  | 50th Percentile | 105.24 | 0.13 | (0.13) | 0.25 | (0.24) | 0.15 | (0.15) | 0.11 | (0.10) |
|  | 75th Percentile | 121.24 | −0.09 | (0.07) | −0.17 | (0.14) | −0.12 | (0.10) | −0.13 | (0.11) |
|  | 90th Percentile | 137.26 | −0.29 | (0.22) | −0.45 | (0.34) | −0.45 | (0.34) | −0.34 | (0.26) |
|  | 95th Percentile | 147.63 | −0.40 | (0.28) | −0.54 | (0.38) | −0.43 | (0.30) | −0.47 | (0.33) |
| X ($\sigma_{u}^{2}=0.3, n = 300$ and $r_{var}=9$) | Average | 116.95 | 0.09 | (0.09) | 0.09 | (0.09) | 0.08 | (0.08) | 0.08 | (0.08) |
|  | 5th Percentile | 81.07 | 0.64 | (0.77) | 2.85 | (3.41) | 0.65 | (0.78) | 0.66 | (0.79) |
|  | 10th Percentile | 87.75 | 0.51 | (0.58) | 2.31 | (2.64) | 0.51 | (0.58) | 0.52 | (0.59) |
|  | 25th Percentile | 99.90 | 0.29 | (0.31) | 1.33 | (1.39) | 0.33 | (0.34) | 0.29 | (0.31) |
|  | 50th Percentile | 114.97 | 0.07 | (0.07) | 0.19 | (0.18) | 0.08 | (0.07) | 0.06 | (0.06) |
|  | 75th Percentile | 131.83 | −0.13 | (0.11) | −1.09 | (0.96) | −0.18 | (0.16) | −0.15 | (0.14) |
|  | 90th Percentile | 148.68 | −0.28 | (0.23) | −2.20 | (1.80) | −0.39 | (0.32) | −0.32 | (0.26) |
|  | 95th Percentile | 159.56 | −0.36 | (0.28) | −2.83 | (2.21) | −0.39 | (0.30) | −0.41 | (0.32) |
| XI ($\sigma_{u}^{2}=0.3, n = 500$ and $r_{var}=4$) | Average | 107.17 | 0.03 | (0.03) | 0.11 | (0.1) | 0.04 | (0.04) | 0.04 | (0.04) |
|  | 5th Percentile | 73.26 | 0.34 | (0.45) | 0.25 | (0.34) | 0.31 | (0.41) | 0.35 | (0.47) |
|  | 10th Percentile | 79.54 | 0.28 | (0.35) | 0.22 | (0.28) | 0.26 | (0.32) | 0.29 | (0.36) |
|  | 25th Percentile | 90.99 | 0.17 | (0.19) | 0.19 | (0.21) | 0.18 | (0.20) | 0.18 | (0.20) |
|  | 50th Percentile | 105.24 | 0.04 | (0.04) | 0.20 | (0.19) | 0.06 | (0.05) | 0.05 | (0.05) |
|  | 75th Percentile | 121.24 | −0.09 | (0.08) | −0.04 | (0.03) | −0.06 | (0.05) | −0.09 | (0.07) |
|  | 90th Percentile | 137.26 | −0.22 | (0.17) | 0.00 | (0.00) | −0.22 | (0.17) | −0.21 | (0.16) |
|  | 95th Percentile | 147.63 | −0.29 | (0.21) | −0.06 | (0.04) | −0.34 | (0.24) | −0.28 | (0.20) |
| XII ($\sigma_{u}^{2}=0.3, n = 500$ and $r_{var}=9$) | Average | 116.95 | 0.04 | (0.04) | 0.06 | (0.06) | 0.03 | (0.03) | 0.03 | (0.03) |
|  | 5th Percentile | 81.07 | 0.65 | (0.78) | 2.00 | (2.40) | 0.63 | (0.75) | 0.65 | (0.78) |
|  | 10th Percentile | 87.75 | 0.52 | (0.59) | 1.59 | (1.82) | 0.52 | (0.59) | 0.52 | (0.59) |
|  | 25th Percentile | 99.90 | 0.29 | (0.3) | 0.90 | (0.95) | 0.29 | (0.30) | 0.29 | (0.30) |
|  | 50th Percentile | 114.97 | 0.04 | (0.03) | 0.14 | (0.14) | 0.03 | (0.03) | 0.03 | (0.03) |
|  | 75th Percentile | 131.83 | −0.22 | (0.19) | −0.79 | (0.70) | −0.21 | (0.18) | −0.22 | (0.20) |
|  | 90th Percentile | 148.68 | −0.44 | (0.36) | −1.51 | (1.23) | −0.46 | (0.37) | −0.44 | (0.36) |
|  | 95th Percentile | 159.56 | −0.56 | (0.44) | −1.99 | (1.55) | −0.61 | (0.47) | −0.57 | (0.45) |

ISU: Iowa State University; NCI: National Cancer Institute; MSM: Multiple Source Method; SPADE: Statistical Program to Assess Dietary Exposure.

Table S3 shows the MSE’s for the four methods in each scenario. The MSE of the estimates for the mean habitual intake agrees for NCI, MSM and SPADE, but is much larger $(60\%-80\%)$ for the ISU method. For the presented percentiles, the MSE of ISU, MSM and SPADE methods are similar, but are the highest for ISU and the lowest for SPADE. The MSE for the percentiles of the NCI method shows greater MSE than the others—except for the scenario III (with $\sigma_{\varepsilon}^{2}=1,n=300$ and $r_{var}=4$),
V (with $\sigma_{\varepsilon}^{2}=1 , n=500$ and $r_{var}=4$), IX and XI (with $\sigma_{u}^{2}=0.3, n=500$ and $r_{var}=4$).

The MSE of the mean did agree for ISU, NCI, MSM and SPADE, whereas the MSE of the percentiles corresponded more for the ISU, MSM and SPADE, but was as twice high for the ISU method. The ISU, MSM and SPADE methods yielded more precise estimates for the percentiles than the NCI method, particularly for the 5th and 95th percentiles when sample size was small and variance ratio was large.

Table S3. MSEs of estimates obtained with each method for each scenario $\left( \boldsymbol{\lambda}\boldsymbol{=0.2} \right)$.

| **Scenario** | **Parameter** | **Method** | | | |
| --- | --- | --- | --- | --- | --- |
|  |  | **ISU** | **NCI** | **MSM** | **SPADE** |
| I  ($\sigma_{\varepsilon}^{2}=1, n = 150$ and $r_{var}=4$) | Average | 16.61 | 9.21 | 9.03 | 9.04 |
|  | 5th Percentile | 47.89 | 69.44 | 46.81 | 44.01 |
|  | 10th Percentile | 37.11 | 49.46 | 35.07 | 32.87 |
|  | 25th Percentile | 22.40 | 22.71 | 18.48 | 17.20 |
|  | 50th Percentile | 16.13 | 9.18 | 10.61 | 9.00 |
|  | 75th Percentile | 29.92 | 23.68 | 21.11 | 19.30 |
|  | 90th Percentile | 71.44 | 74.97 | 58.29 | 55.47 |
|  | 95th Percentile | 115.90 | 130.85 | 104.79 | 95.16 |
| II  ($\sigma_{\varepsilon}^{2}=1, n = 150$ and $r_{var}=9$) | Average | 14.28 | 7.36 | 7.27 | 7.28 |
|  | 5th Percentile | 56.06 | 115.26 | 52.35 | 51.02 |
|  | 10th Percentile | 41.49 | 76.79 | 37.23 | 36.30 |
|  | 25th Percentile | 22.57 | 29.54 | 17.62 | 16.87 |
|  | 50th Percentile | 14.12 | 7.35 | 7.99 | 7.29 |
|  | 75th Percentile | 26.45 | 28.89 | 18.10 | 17.56 |
|  | 90th Percentile | 64.02 | 98.66 | 53.01 | 51.95 |
|  | 95th Percentile | 102.78 | 170.06 | 93.18 | 87.92 |
| III  ($\sigma_{\varepsilon}^{2}=1, n = 300$ and $r_{var}=4$) | Average | 8.22 | 4.93 | 4.91 | 4.91 |
|  | 5th Percentile | 22.73 | 25.53 | 21.09 | 20.29 |
|  | 10th Percentile | 17.91 | 18.85 | 16.64 | 15.40 |
|  | 25th Percentile | 11.15 | 9.60 | 9.27 | 8.43 |
|  | 50th Percentile | 8.06 | 4.90 | 5.41 | 4.84 |
|  | 75th Percentile | 14.18 | 10.83 | 10.43 | 9.94 |
|  | 90th Percentile | 33.25 | 31.28 | 29.62 | 27.39 |
|  | 95th Percentile | 53.79 | 53.58 | 51.59 | 46.50 |
| IV  ($\sigma_{\varepsilon}^{2}=1, n = 300$ and $r_{var}=9$) | Average | 7.21 | 3.91 | 3.91 | 3.91 |
|  | 5th Percentile | 36.24 | 97.72 | 34.48 | 33.87 |
|  | 10th Percentile | 26.20 | 64.35 | 24.21 | 23.70 |
|  | 25th Percentile | 13.21 | 23.13 | 10.77 | 10.43 |
|  | 50th Percentile | 7.19 | 4.00 | 4.19 | 3.93 |
|  | 75th Percentile | 14.57 | 21.16 | 10.89 | 10.53 |
|  | 90th Percentile | 37.60 | 76.33 | 33.39 | 32.51 |
|  | 95th Percentile | 61.15 | 131.58 | 57.57 | 55.21 |
| V  ($\sigma_{\varepsilon}^{2}=1, n = 500$ and $r_{var}=4$) | Average | 4.56 | 2.85 | 2.83 | 2.84 |
|  | 5th Percentile | 12.89 | 12.70 | 12.93 | 11.90 |
|  | 10th Percentile | 10.11 | 9.57 | 9.56 | 9.04 |
|  | 25th Percentile | 6.21 | 5.11 | 5.25 | 4.93 |
|  | 50th Percentile | 4.47 | 2.84 | 3.30 | 2.80 |
|  | 75th Percentile | 8.14 | 5.99 | 6.32 | 5.85 |
|  | 90th Percentile | 19.44 | 17.01 | 17.86 | 16.27 |
|  | 95th Percentile | 31.58 | 28.98 | 30.47 | 27.70 |
| VI  ($\sigma_{\varepsilon}^{2}=1, n = 500$ and $r_{var}=9$) | Average | 3.99 | 2.30 | 2.29 | 2.29 |
|  | 5th Percentile | 26.13 | 64.47 | 24.83 | 24.35 |
|  | 10th Percentile | 18.57 | 42.39 | 17.07 | 16.84 |
|  | 25th Percentile | 8.76 | 14.99 | 7.24 | 7.08 |
|  | 50th Percentile | 4.01 | 2.34 | 2.50 | 2.31 |
|  | 75th Percentile | 8.87 | 13.99 | 7.21 | 7.04 |
|  | 90th Percentile | 24.82 | 51.25 | 23.62 | 22.76 |
|  | 95th Percentile | 41.18 | 88.12 | 40.06 | 38.91 |

**Table S3.** *Cont*.

| **Scenario** | **Parameter** | **Method** | | | |
| --- | --- | --- | --- | --- | --- |
|  |  | **ISU** | **NCI** | **MSM** | **SPADE** |
| VII  ($\sigma_{u}^{2}=0.3 , n = 150$ and $r_{var}=4$) | Average | 20.57 | 11.39 | 11.15 | 11.18 |
|  | 5th Percentile | 56.31 | 85.21 | 55.09 | 51.85 |
|  | 10th Percentile | 44.13 | 61.04 | 41.74 | 39.19 |
|  | 25th Percentile | 27.16 | 28.24 | 22.43 | 20.94 |
|  | 50th Percentile | 19.86 | 11.33 | 13.06 | 11.10 |
|  | 75th Percentile | 37.15 | 29.67 | 26.01 | 23.80 |
|  | 90th Percentile | 89.91 | 95.83 | 72.95 | 69.46 |
|  | 95th Percentile | 147.20 | 168.87 | 132.75 | 120.41 |
| VIII  ($\sigma_{u}^{2}=0.3, n = 150$ and $r_{var}=9$) | Average | 48.57 | 25.04 | 24.67 | 24.69 |
|  | 5th Percentile | 144.83 | 196.04 | 135.88 | 133.34 |
|  | 10th Percentile | 113.67 | 140.20 | 102.66 | 100.74 |
|  | 25th Percentile | 68.78 | 64.91 | 54.84 | 52.51 |
|  | 50th Percentile | 47.03 | 24.64 | 26.82 | 24.68 |
|  | 75th Percentile | 89.07 | 69.70 | 58.24 | 56.23 |
|  | 90th Percentile | 227.00 | 235.31 | 183.51 | 178.16 |
|  | 95th Percentile | 380.23 | 422.22 | 341.75 | 317.05 |
| IX  ($\sigma_{u}^{2}=0.3 , n = 300$ and $r_{var}=4$) | Average | 10.16 | 6.09 | 6.08 | 6.08 |
|  | 5th Percentile | 26.66 | 30.99 | 24.82 | 23.86 |
|  | 10th Percentile | 21.27 | 23.05 | 19.78 | 18.34 |
|  | 25th Percentile | 13.51 | 11.86 | 11.27 | 10.26 |
|  | 50th Percentile | 9.93 | 6.05 | 6.66 | 5.97 |
|  | 75th Percentile | 17.61 | 13.51 | 12.89 | 12.27 |
|  | 90th Percentile | 41.84 | 39.77 | 37.18 | 34.32 |
|  | 95th Percentile | 68.32 | 68.79 | 65.39 | 58.86 |
| X  ($\sigma_{u}^{2}=0.3, n = 300$ and $r_{var}=9$) | Average | 24.23 | 13.16 | 13.18 | 13.14 |
|  | 5th Percentile | 96.20 | 135.90 | 92.36 | 90.74 |
|  | 10th Percentile | 73.36 | 96.18 | 68.78 | 67.09 |
|  | 25th Percentile | 40.76 | 41.99 | 34.10 | 32.82 |
|  | 50th Percentile | 23.93 | 13.36 | 14.13 | 13.31 |
|  | 75th Percentile | 48.03 | 42.08 | 34.77 | 33.29 |
|  | 90th Percentile | 130.52 | 146.67 | 114.73 | 110.18 |
|  | 95th Percentile | 220.99 | 261.10 | 207.17 | 196.09 |
| XI  ($\sigma_{u}^{2}=0.3 , n = 500$ and $r_{var}=4$) | Average | 5.63 | 3.52 | 3.50 | 3.50 |
|  | 5th Percentile | 15.11 | 15.72 | 15.14 | 13.98 |
|  | 10th Percentile | 12.00 | 11.90 | 11.35 | 10.76 |
|  | 25th Percentile | 7.53 | 6.37 | 6.39 | 6.00 |
|  | 50th Percentile | 5.49 | 3.49 | 4.05 | 3.45 |
|  | 75th Percentile | 10.08 | 7.54 | 7.80 | 7.20 |
|  | 90th Percentile | 24.40 | 22.00 | 22.38 | 20.36 |
|  | 95th Percentile | 40.03 | 37.89 | 38.52 | 35.02 |
| XII  ($\sigma_{u}^{2}=0.3 , n = 500$ and $r_{var}=9$) | Average | 13.08 | 7.59 | 7.59 | 7.56 |
|  | 5th Percentile | 70.47 | 96.46 | 66.91 | 65.90 |
|  | 10th Percentile | 52.73 | 67.92 | 48.78 | 48.06 |
|  | 25th Percentile | 27.33 | 28.77 | 23.03 | 22.39 |
|  | 50th Percentile | 13.24 | 7.79 | 8.33 | 7.75 |
|  | 75th Percentile | 28.20 | 27.44 | 22.46 | 21.74 |
|  | 90th Percentile | 83.91 | 100.82 | 79.86 | 76.05 |
|  | 95th Percentile | 145.38 | 179.87 | 141.74 | 136.32 |

ISU, Iowa State University; NCI, National Cancer Institute; MSM, Multiple Source Method; SPADE, Statistical Program to Assess Dietary Exposure.

Figure S1 show boxplots of the biases in each scenario, confirming similar results between the methods. However, it is clear that all methods were less accurate for estimation of the 5th and 95th percentiles across all scenarios. As expected, accuracy was lower in scenarios with a smaller sample size $(n=150)$. It is interesting to see that in the first six scenarios with between-person variance equal to one, the differences in the spread of the bias are much less than for the scenarios VII-XII.


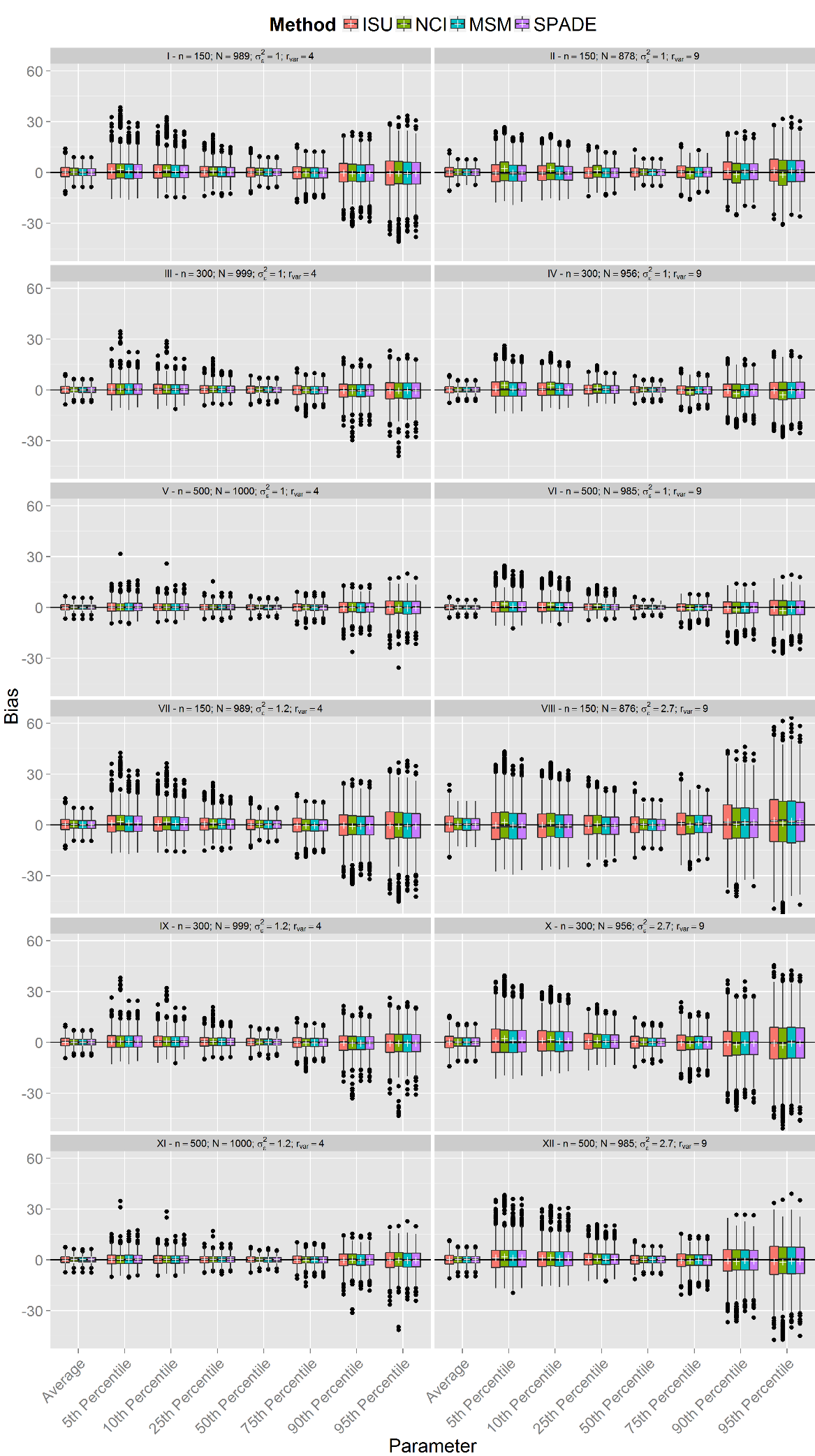


**Figure S1.** Boxplot of biases calculated for each method and scenarios.

1. **Results per Method without Excluded Samples when Others Method Estimated
   between-Person Variance Equal to Zero**

Table S4 shows the true value, bias and relative bias for the mean and some percentiles of the usual intake distribution in each scenario per method without excluding samples when others methods estimated between-person variance equal to zero. As the table shows, the compared methods behaved similarly, yielding estimates close to the true values; although the NCI method showed the best estimates for the scenario VIII in the results when at least one method estimated between-person variance equal to zero (results in Tables 1 and 2 in the paper). Because of this, the NCI method may have benefitted by the exclusion of those samples.

**Table S4.** Bias and relative bias of estimates obtained with each method for each scenario $\left( \lambda=0.2 \right)$.

| **Scenario** | **Parameter** | **True Value** | **Bias (Relative Bias %)** | | | | | | | |
| --- | --- | --- | --- | --- | --- | --- | --- | --- | --- | --- |
|  |  |  | **ISU** | | **NCI** | | **MSM** | | **SPADE** | |
| I ($\sigma_{\varepsilon}^{2}=1 , n = 150$ and $r_{var}=4$) | Average | 105.56 | 0.21 | (0.20) | 0.35 | (0.33) | 0.11 | (0.11) | 0.13 | (0.12) |
|  | 5th Percentile | 74.64 | 0.91 | (1.21) | 2.06 | (2.76) | 1.28 | (1.71) | 1.16 | (1.55) |
|  | 10th Percentile | 80.46 | 0.74 | (0.92) | 1.80 | (2.24) | 1.05 | (1.30) | 0.94 | (1.16) |
|  | 25th Percentile | 90.99 | 0.47 | (0.52) | 1.04 | (1.14) | 0.59 | (0.65) | 0.56 | (0.61) |
|  | 50th Percentile | 103.97 | 0.18 | (0.17) | 0.44 | (0.42) | 0.18 | (0.17) | 0.12 | (0.12) |
|  | 75th Percentile | 118.39 | −0.09 | (0.07) | −0.43 | (0.37) | −0.4 | (0.34) | −0.31 | (0.26) |
|  | 90th Percentile | 132.71 | −0.28 | (0.21) | −0.94 | (0.71) | −0.85 | (0.64) | −0.67 | (0.51) |
|  | 95th Percentile | 141.93 | −0.37 | (0.26) | −1.08 | (0.76) | −0.97 | (0.68) | −0.88 | (0.62) |
| II ($\sigma_{\varepsilon}^{2}=1 , n = 150$ and $r_{var}=9$) | Average | 104.67 | 0.23 | (0.22) | 0.23 | (0.22) | 0.07 | (0.07) | 0.12 | (0.12) |
|  | 5th Percentile | 83.56 | −0.19 | (0.23) | 4.48 | (5.36) | 2.06 | (2.46) | 1.47 | (1.76) |
|  | 10th Percentile | 87.76 | −0.17 | (0.20) | 3.63 | (4.13) | 1.58 | (1.80) | 1.14 | (1.30) |
|  | 25th Percentile | 95.16 | −0.09 | (0.10) | 1.99 | (2.09) | 0.82 | (0.86) | 0.61 | (0.64) |
|  | 50th Percentile | 103.97 | 0.1 | (0.10) | 0.31 | (0.30) | 0.08 | (0.07) | 0.06 | (0.06) |
|  | 75th Percentile | 113.41 | 0.41 | (0.36) | −1.54 | (1.36) | −0.73 | (0.64) | −0.43 | (0.38) |
|  | 90th Percentile | 122.5 | 0.81 | (0.66) | −3.06 | (2.50) | −1.46 | (1.20) | −0.82 | (0.67) |
|  | 95th Percentile | 128.21 | 1.12 | (0.87) | −3.88 | (3.03) | −1.79 | (1.40) | −1.02 | (0.79) |
| III ($\sigma_{\varepsilon}^{2}=1 , n = 300$ and $r_{var}=4$) | Average | 105.56 | 0.12 | (0.11) | 0.13 | (0.12) | 0.09 | (0.08) | 0.1 | (0.09) |
|  | 5th Percentile | 74.64 | 0.62 | (0.82) | 0.66 | (0.88) | 0.61 | (0.81) | 0.65 | (0.88) |
|  | 10th Percentile | 80.46 | 0.52 | (0.64) | 0.64 | (0.79) | 0.52 | (0.64) | 0.54 | (0.68) |
|  | 25th Percentile | 90.99 | 0.34 | (0.37) | 0.47 | (0.52) | 0.36 | (0.4) | 0.35 | (0.38) |
|  | 50th Percentile | 103.97 | 0.13 | (0.13) | 0.26 | (0.25) | 0.16 | (0.15) | 0.11 | (0.10) |
|  | 75th Percentile | 118.39 | −0.09 | (0.07) | −0.18 | (0.15) | −0.13 | (0.11) | −0.14 | (0.12) |
|  | 90th Percentile | 132.71 | −0.29 | (0.22) | −0.48 | (0.36) | −0.47 | (0.36) | −0.37 | (0.28) |
|  | 95th Percentile | 141.93 | −0.4 | (0.28) | −0.57 | (0.40) | −0.46 | (0.32) | −0.50 | (0.35) |
| IV ($\sigma_{\varepsilon}^{2}=1 , n = 300$ and $r_{var}=9$) | Average | 104.67 | 0.09 | (0.09) | 0.10 | (0.10) | 0.08 | (0.08) | 0.09 | (0.08) |
|  | 5th Percentile | 83.56 | 0.64 | (0.77) | 3.65 | (4.37) | 1.42 | (1.70) | 1.25 | (1.49) |
|  | 10th Percentile | 87.76 | 0.51 | (0.58) | 2.96 | (3.37) | 1.13 | (1.29) | 0.99 | (1.13) |
|  | 25th Percentile | 95.16 | 0.29 | (0.31) | 1.69 | (1.78) | 0.68 | (0.72) | 0.56 | (0.59) |
|  | 50th Percentile | 103.97 | 0.07 | (0.07) | 0.23 | (0.22) | 0.11 | (0.11) | 0.08 | (0.08) |
|  | 75th Percentile | 113.41 | −0.13 | (0.11) | −1.40 | (1.24) | −0.50 | (0.44) | −0.39 | (0.34) |
|  | 90th Percentile | 122.5 | −0.28 | (0.23) | −2.86 | (2.34) | −1.04 | (0.85) | −0.81 | (0.66) |
|  | 95th Percentile | 128.21 | −0.36 | (0.28) | −3.70 | (2.89) | −1.27 | (0.99) | −1.05 | (0.82) |
| V ($\sigma_{\varepsilon}^{2}=1 , n = 500$ and $r_{var}=4$) | Average | 105.56 | 0.03 | (0.03) | 0.11 | (0.10) | 0.04 | (0.04) | 0.04 | (0.04) |
|  | 5th Percentile | 74.64 | 0.34 | (0.45) | 0.25 | (0.34) | 0.31 | (0.41) | 0.35 | (0.47) |
|  | 10th Percentile | 80.46 | 0.28 | (0.35) | 0.22 | (0.28) | 0.26 | (0.32) | 0.29 | (0.36) |
|  | 25th Percentile | 90.99 | 0.17 | (0.19) | 0.19 | (0.21) | 0.18 | (0.20) | 0.18 | (0.20) |
|  | 50th Percentile | 103.97 | 0.04 | (0.04) | 0.20 | (0.19) | 0.06 | (0.05) | 0.05 | (0.05) |
|  | 75th Percentile | 118.39 | −0.09 | (0.08) | −0.04 | (0.03) | −0.06 | (0.05) | −0.09 | (0.07) |
|  | 90th Percentile | 132.71 | −0.22 | (0.17) | 0.00 | (0.00) | −0.22 | (0.17) | −0.21 | (0.16) |
|  | 95th Percentile | 141.93 | −0.29 | (0.21) | −0.06 | (0.04) | −0.34 | (0.24) | −0.28 | (0.20) |

**Table S4.** *Cont*.

| **Scenario** | **Parameter** | **True Value** | **Bias (Relative Bias %)** | | | | | | | |
| --- | --- | --- | --- | --- | --- | --- | --- | --- | --- | --- |
|  |  |  | **ISU** | | **NCI** | | **MSM** | | **SPADE** | |
| VI ($\sigma_{\varepsilon}^{2}=1 , n = 500$ and $r_{var}=9$) | Average | 104.67 | 0.04 | (0.04) | 0.06 | (0.06) | 0.03 | (0.03) | 0.03 | (0.03) |
|  | 5th Percentile | 83.56 | 0.65 | (0.78) | 2.27 | (2.72) | 0.89 | (1.06) | 0.89 | (1.07) |
|  | 10th Percentile | 87.76 | 0.52 | (0.59) | 1.81 | (2.06) | 0.73 | (0.83) | 0.71 | (0.81) |
|  | 25th Percentile | 95.16 | 0.29 | (0.30) | 1.03 | (1.08) | 0.41 | (0.43) | 0.39 | (0.41) |
|  | 50th Percentile | 103.97 | 0.04 | (0.03) | 0.15 | (0.14) | 0.04 | (0.04) | 0.04 | (0.03) |
|  | 75th Percentile | 113.41 | −0.22 | (0.19) | −0.90 | (0.80) | −0.32 | (0.28) | −0.33 | (0.29) |
|  | 90th Percentile | 122.5 | −0.44 | (0.36) | −1.73 | (1.42) | −0.69 | (0.56) | −0.65 | (0.53) |
|  | 95th Percentile | 128.21 | −0.56 | (0.44) | −2.29 | (1.79) | −0.91 | (0.71) | −0.85 | (0.66) |
| VII ($\sigma_{u}^{2}=0.3 , n = 150$ and $r_{var}=4$) | Average | 107.17 | 0.24 | (0.23) | 0.39 | (0.36) | 0.13 | (0.12) | 0.15 | (0.14) |
|  | 5th Percentile | 73.26 | 1.00 | (1.37) | 2.35 | (3.20) | 1.4 | (1.91) | 1.24 | (1.70) |
|  | 10th Percentile | 79.54 | 0.83 | (1.04) | 2.05 | (2.58) | 1.14 | (1.44) | 1.01 | (1.27) |
|  | 25th Percentile | 90.99 | 0.53 | (0.58) | 1.18 | (1.30) | 0.64 | (0.71) | 0.6 | (0.66) |
|  | 50th Percentile | 105.24 | 0.21 | (0.20) | 0.49 | (0.47) | 0.20 | (0.19) | 0.14 | (0.13) |
|  | 75th Percentile | 121.24 | −0.08 | (0.07) | −0.50 | (0.42) | −0.42 | (0.35) | −0.32 | (0.26) |
|  | 90th Percentile | 137.26 | −0.29 | (0.21) | −1.09 | (0.79) | −0.92 | (0.67) | −0.7 | (0.51) |
|  | 95th Percentile | 147.63 | −0.38 | (0.26) | −1.26 | (0.85) | −1.05 | (0.71) | −0.9 | (0.61) |
| VIII  ($\sigma_{u}^{2}=0.3 , n = 150$ and $r_{var}=9$) | Average | 116.95 | 0.52 | (0.44) | 0.44 | (0.38) | 0.14 | (0.12) | 0.23 | (0.19) |
|  | 5th Percentile | 81.07 | −0.18 | (0.22) | 5.72 | (7.05) | 3.52 | (4.35) | 2.56 | (3.16) |
|  | 10th Percentile | 87.75 | −0.23 | (0.26) | 4.68 | (5.33) | 2.63 | (2.99) | 1.96 | (2.24) |
|  | 25th Percentile | 99.9 | −0.18 | (0.18) | 2.57 | (2.57) | 1.29 | (1.29) | 1.01 | (1.01) |
|  | 50th Percentile | 114.97 | 0.14 | (0.12) | 0.50 | (0.44) | 0.1 | (0.09) | 0.05 | (0.04) |
|  | 75th Percentile | 131.83 | 0.8 | (0.6) | −1.82 | (1.38) | −1.16 | (0.88) | −0.76 | (0.57) |
|  | 90th Percentile | 148.68 | 1.76 | (1.18) | −3.55 | (2.39) | −2.34 | (1.57) | −1.29 | (0.87) |
|  | 95th Percentile | 159.56 | 2.53 | (1.59) | −4.37 | (2.74) | −2.8 | (1.76) | −1.5 | (0.94) |
| IX ($\sigma_{u}^{2}=0.3 , n = 300$ and $r_{var}=4$) | Average | 107.17 | 0.14 | (0.13) | 0.14 | (0.13) | 0.1 | (0.1) | 0.11 | (0.1) |
|  | 5th Percentile | 73.26 | 0.68 | (0.93) | 0.74 | (1.01) | 0.66 | (0.9) | 0.72 | (0.98) |
|  | 10th Percentile | 79.54 | 0.57 | (0.72) | 0.71 | (0.90) | 0.55 | (0.69) | 0.6 | (0.75) |
|  | 25th Percentile | 90.99 | 0.38 | (0.42) | 0.53 | (0.58) | 0.38 | (0.42) | 0.38 | (0.42) |
|  | 50th Percentile | 105.24 | 0.15 | (0.14) | 0.29 | (0.27) | 0.17 | (0.16) | 0.12 | (0.12) |
|  | 75th Percentile | 121.24 | −0.09 | (0.07) | −0.20 | (0.16) | −0.12 | (0.10) | −0.15 | (0.12) |
|  | 90th Percentile | 137.26 | −0.31 | (0.22) | −0.54 | (0.39) | −0.49 | (0.36) | −0.4 | (0.29) |
|  | 95th Percentile | 147.63 | −0.43 | (0.29) | −0.64 | (0.43) | −0.47 | (0.32) | −0.54 | (0.37) |
| X ($\sigma_{u}^{2}=0.3 , n = 300$ and $r_{var}=9$) | Average | 116.95 | 0.22 | (0.19) | 0.21 | (0.18) | 0.19 | (0.17) | 0.16 | (0.14) |
|  | 5th Percentile | 81.07 | 1.19 | (1.47) | 3.53 | (4.36) | 2.43 | (3.00) | 2.16 | (2.67) |
|  | 10th Percentile | 87.75 | 0.95 | (1.08) | 2.93 | (3.34) | 1.88 | (2.14) | 1.73 | (1.97) |
|  | 25th Percentile | 99.90 | 0.55 | (0.55) | 1.76 | (1.76) | 1.12 | (1.12) | 0.99 | (0.99) |
|  | 50th Percentile | 114.97 | 0.15 | (0.13) | 0.39 | (0.34) | 0.23 | (0.20) | 0.15 | (0.13) |
|  | 75th Percentile | 131.83 | −0.19 | (0.14) | −1.26 | (0.95) | −0.7 | (0.53) | −0.67 | (0.51) |
|  | 90th Percentile | 148.68 | −0.41 | (0.28) | −2.67 | (1.79) | −1.59 | (1.07) | −1.39 | (0.93) |
|  | 95th Percentile | 159.56 | −0.5 | (0.31) | −3.41 | (2.14) | −1.98 | (1.24) | −1.8 | (1.12) |
| XI ($\sigma_{u}^{2}=0.3 , n = 500$ and $r_{var}=4$) | Average | 107.17 | 0.04 | (0.04) | 0.12 | (0.11) | 0.05 | (0.04) | 0.05 | (0.05) |
|  | 5th Percentile | 73.26 | 0.37 | (0.51) | 0.30 | (0.41) | 0.34 | (0.46) | 0.39 | (0.53) |
|  | 10th Percentile | 79.54 | 0.31 | (0.39) | 0.26 | (0.33) | 0.28 | (0.35) | 0.32 | (0.40) |
|  | 25th Percentile | 90.99 | 0.19 | (0.21) | 0.21 | (0.23) | 0.19 | (0.21) | 0.20 | (0.22) |
|  | 50th Percentile | 105.24 | 0.05 | (0.05) | 0.21 | (0.20) | 0.06 | (0.06) | 0.06 | (0.06) |
|  | 75th Percentile | 121.24 | −0.1 | (0.08) | −0.06 | (0.05) | −0.05 | (0.04) | −0.09 | (0.07) |
|  | 90th Percentile | 137.26 | −0.24 | (0.17) | −0.03 | (0.02) | −0.22 | (0.16) | −0.23 | (0.17) |
|  | 95th Percentile | 147.63 | −0.32 | (0.22) | −0.09 | (0.06) | −0.36 | (0.25) | −0.31 | (0.21) |
| XII ($\sigma_{u}^{2}=0.3 , n = 500$ and $r_{var}=9$) | Average | 116.95 | 0.10 | (0.09) | 0.13 | (0.12) | 0.10 | (0.09) | 0.08 | (0.07) |
|  | 5th Percentile | 81.07 | 1.17 | (1.45) | 2.17 | (2.68) | 1.50 | (1.85) | 1.50 | (1.85) |
|  | 10th Percentile | 87.75 | 0.94 | (1.07) | 1.75 | (2.00) | 1.16 | (1.32) | 1.20 | (1.37) |
|  | 25th Percentile | 99.90 | 0.54 | (0.54) | 1.04 | (1.04) | 0.61 | (0.62) | 0.68 | (0.68) |
|  | 50th Percentile | 114.97 | 0.10 | (0.08) | 0.26 | (0.23) | 0.10 | (0.09) | 0.09 | (0.08) |
|  | 75th Percentile | 131.83 | −0.34 | (0.26) | −0.81 | (0.62) | −0.38 | (0.29) | −0.52 | (0.39) |
|  | 90th Percentile | 148.68 | −0.72 | (0.48) | −1.52 | (1.03) | −0.93 | (0.63) | −1.06 | (0.71) |
|  | 95th Percentile | 159.56 | −0.93 | (0.58) | −2.04 | (1.28) | −1.33 | (0.84) | −1.37 | (0.86) |

ISU: Iowa State University; NCI: National Cancer Institute; MSM: Multiple Source Method; SPADE: Statistical Program to Assess Dietary Exposure.

Table S5 shows the MSE for the mean and some percentiles of the usual intake distribution in each scenario for all samples. The NCI method showed greater MSE than the others—except for the scenario V (with $\sigma_{\varepsilon}^{2}=1 , n=500$ and $r_{var}=4$) and XI (with $\sigma_{u}^{2}=0.3, n=500$ and $r_{var}=4$). The SPADE method showed the smaller MSE than others for the major part of scenarios.

**Table S5.** MSEs of estimates obtained with each method for each scenario $\left( \lambda=0.2 \right)$.

| **Scenario** | **Parameter** | **Method** | | | |
| --- | --- | --- | --- | --- | --- |
|  |  | **ISU** | **NCI** | **MSM** | **SPADE** |
| I  ($\sigma_{\varepsilon}^{2}=1 , n = 150$ and $r_{var}=4$) | Average | 16.6 | 9.14 | 9.0 | 9.0 |
|  | 5th Percentile | 47.9 | 78.41 | 54.9 | 50.7 |
|  | 10th Percentile | 37.1 | 55.35 | 40.4 | 37.3 |
|  | 25th Percentile | 22.4 | 24.67 | 20.3 | 18.7 |
|  | 50th Percentile | 16.1 | 9.15 | 10.6 | 9.0 |
|  | 75th Percentile | 29.9 | 25.09 | 22.4 | 20.2 |
|  | 90th Percentile | 71.4 | 81.57 | 64.3 | 60.0 |
|  | 95th Percentile | 115.9 | 142.74 | 115.6 | 103.5 |
| II  ($\sigma_{\varepsilon}^{2}=1 , n = 150$ and $r_{var}=9$) | Average | 14.3 | 7.41 | 7.3 | 7.3 |
|  | 5th Percentile | 56.1 | 154.15 | 86.2 | 82.6 |
|  | 10th Percentile | 41.5 | 101.88 | 58.9 | 56.6 |
|  | 25th Percentile | 22.6 | 37.65 | 24.4 | 23.4 |
|  | 50th Percentile | 14.1 | 7.45 | 8.0 | 7.4 |
|  | 75th Percentile | 26.5 | 34.64 | 24.1 | 22.5 |
|  | 90th Percentile | 64.0 | 123.21 | 77.5 | 72.5 |
|  | 95th Percentile | 102.8 | 212.81 | 135.1 | 123.7 |
| III  ($\sigma_{\varepsilon}^{2}=1 , n = 300$ and $r_{var}=4$) | Average | 8.2 | 4.92 | 4.9 | 4.9 |
|  | 5th Percentile | 22.7 | 26.53 | 21.9 | 21.3 |
|  | 10th Percentile | 17.9 | 19.52 | 17.2 | 16.1 |
|  | 25th Percentile | 11.2 | 9.83 | 9.5 | 8.7 |
|  | 50th Percentile | 8.1 | 4.90 | 5.4 | 4.8 |
|  | 75th Percentile | 14.2 | 10.96 | 10.5 | 10.1 |
|  | 90th Percentile | 33.2 | 31.93 | 30.1 | 28.0 |
|  | 95th Percentile | 53.8 | 54.78 | 52.6 | 47.7 |
| IV  ($\sigma_{\varepsilon}^{2}=1 , n = 300$ and $r_{var}=9$) | Average | 7.2 | 3.94 | 3.9 | 3.9 |
|  | 5th Percentile | 36.2 | 113.55 | 47.7 | 45.9 |
|  | 10th Percentile | 26.2 | 74.61 | 32.8 | 31.5 |
|  | 25th Percentile | 13.2 | 26.52 | 13.6 | 12.9 |
|  | 50th Percentile | 7.2 | 4.07 | 4.2 | 3.9 |
|  | 75th Percentile | 14.6 | 23.47 | 13.0 | 12.4 |
|  | 90th Percentile | 37.6 | 86.38 | 42.4 | 40.4 |
|  | 95th Percentile | 61.1 | 149.23 | 73.5 | 69.0 |
| V  ($\sigma_{\varepsilon}^{2}=1 , n = 500$ and $r_{var}=4$) | Average | 4.6 | 2.85 | 2.8 | 2.8 |
|  | 5th Percentile | 12.9 | 12.70 | 12.9 | 11.9 |
|  | 10th Percentile | 10.1 | 9.57 | 9.6 | 9.0 |
|  | 25th Percentile | 6.2 | 5.11 | 5.3 | 4.9 |
|  | 50th Percentile | 4.5 | 2.84 | 3.3 | 2.8 |
|  | 75th Percentile | 8.1 | 5.99 | 6.3 | 5.8 |
|  | 90th Percentile | 19.4 | 17.01 | 17.9 | 16.3 |
|  | 95th Percentile | 31.6 | 28.98 | 30.5 | 27.7 |

**Table S5.** *Cont*.

| **Scenario** | **Parameter** | **Method** | | | |
| --- | --- | --- | --- | --- | --- |
|  |  | **ISU** | **NCI** | **MSM** | **SPADE** |
| VI  ($\sigma_{\varepsilon}^{2}=1 , n = 500$ and $r_{var}=9$) | Average | 4.0 | 2.28 | 2.3 | 2.3 |
|  | 5th Percentile | 26.1 | 69.83 | 29.3 | 29.3 |
|  | 10th Percentile | 18.6 | 45.82 | 19.9 | 20.0 |
|  | 25th Percentile | 8.8 | 16.07 | 8.1 | 8.0 |
|  | 50th Percentile | 4.0 | 2.33 | 2.5 | 2.3 |
|  | 75th Percentile | 8.9 | 14.88 | 8.0 | 7.9 |
|  | 90th Percentile | 24.8 | 54.99 | 27.0 | 26.4 |
|  | 95th Percentile | 41.2 | 94.66 | 46.0 | 45.2 |
| VII  ($\sigma_{u}^{2}=0.3 , n = 150$ and $r_{var}=4$) | Average | 20.6 | 11.31 | 11.1 | 11.1 |
|  | 5th Percentile | 56.3 | 97.22 | 64.7 | 59.0 |
|  | 10th Percentile | 44.1 | 69.03 | 48.2 | 44.0 |
|  | 25th Percentile | 27.2 | 30.98 | 24.6 | 22.6 |
|  | 50th Percentile | 19.9 | 11.31 | 13.0 | 11.1 |
|  | 75th Percentile | 37.1 | 31.46 | 27.5 | 24.7 |
|  | 90th Percentile | 89.9 | 104.55 | 80.3 | 74.1 |
|  | 95th Percentile | 147.2 | 184.77 | 146.2 | 129.0 |
| VIII  ($\sigma_{u}^{2}=0.3 , n = 150$ and $r_{var}=9$) | Average | 48.6 | 25.08 | 24.9 | 24.9 |
|  | 5th Percentile | 144.8 | 332.27 | 233.0 | 222.9 |
|  | 10th Percentile | 113.7 | 230.47 | 166.6 | 159.8 |
|  | 25th Percentile | 68.8 | 95.88 | 75.8 | 72.5 |
|  | 50th Percentile | 47.0 | 25.27 | 26.9 | 25.2 |
|  | 75th Percentile | 89.1 | 90.74 | 76.7 | 71.4 |
|  | 90th Percentile | 227.0 | 331.05 | 263.2 | 244.6 |
|  | 95th Percentile | 380.2 | 593.24 | 481.1 | 435.3 |
| IX  ($\sigma_{u}^{2}=0.3 , n = 300$ and $r_{var}=4$) | Average | 10.2 | 6.09 | 6.1 | 6.1 |
|  | 5th Percentile | 26.7 | 32.19 | 25.8 | 25.1 |
|  | 10th Percentile | 21.3 | 23.86 | 20.4 | 19.2 |
|  | 25th Percentile | 13.5 | 12.15 | 11.5 | 10.6 |
|  | 50th Percentile | 9.9 | 6.05 | 6.7 | 6.0 |
|  | 75th Percentile | 17.6 | 13.66 | 13.0 | 12.4 |
|  | 90th Percentile | 41.8 | 40.57 | 37.8 | 35.1 |
|  | 95th Percentile | 68.3 | 70.27 | 66.6 | 60.3 |
| X  ($\sigma_{u}^{2}=0.3 , n = 300$ and $r_{var}=9$) | Average | 24.2 | 13.26 | 13.3 | 13.2 |
|  | 5th Percentile | 96.2 | 186.96 | 130.2 | 124.2 |
|  | 10th Percentile | 73.4 | 130.26 | 94.1 | 89.1 |
|  | 25th Percentile | 40.8 | 54.01 | 43.1 | 40.2 |
|  | 50th Percentile | 23.9 | 13.72 | 14.4 | 13.4 |
|  | 75th Percentile | 48.0 | 49.68 | 41.0 | 39.3 |
|  | 90th Percentile | 130.5 | 182.49 | 143.6 | 136.5 |
|  | 95th Percentile | 221.0 | 326.06 | 259.7 | 243.0 |
| XI  ($\sigma_{u}^{2}=0.3 , n = 500$ and $r_{var}=4$) | Average | 5.6 | 3.52 | 3.5 | 3.5 |
|  | 5th Percentile | 15.1 | 15.72 | 15.1 | 14.0 |
|  | 10th Percentile | 12.0 | 11.90 | 11.3 | 10.8 |
|  | 25th Percentile | 7.5 | 6.37 | 6.4 | 6.0 |
|  | 50th Percentile | 5.5 | 3.49 | 4.0 | 3.4 |
|  | 75th Percentile | 10.1 | 7.54 | 7.8 | 7.2 |
|  | 90th Percentile | 24.4 | 22.00 | 22.4 | 20.4 |
|  | 95th Percentile | 40.0 | 37.89 | 38.5 | 35.0 |

**Table S5.** *Cont*.

| **Scenario** | **Parameter** | **Method** | | | |
| --- | --- | --- | --- | --- | --- |
|  |  | **ISU** | **NCI** | **MSM** | **SPADE** |
| XII  ($\sigma_{u}^{2}=0.3 , n = 500$ and $r_{var}=9$) | Average | 13.1 | 7.53 | 7.5 | 7.5 |
|  | 5th Percentile | 70.5 | 113.57 | 79.7 | 77.6 |
|  | 10th Percentile | 52.7 | 79.19 | 57.3 | 55.8 |
|  | 25th Percentile | 27.3 | 32.53 | 25.9 | 24.9 |
|  | 50th Percentile | 13.2 | 7.78 | 8.3 | 7.7 |
|  | 75th Percentile | 28.2 | 30.41 | 24.9 | 23.9 |
|  | 90th Percentile | 83.9 | 114.28 | 90.8 | 85.6 |
|  | 95th Percentile | 145.4 | 204.07 | 161.4 | 153.4 |

ISU, Iowa State University, NCI, National Cancer Institute; MSM, Multiple Source Method; SPADE, Statistical Program to Assess Dietary Exposure.
